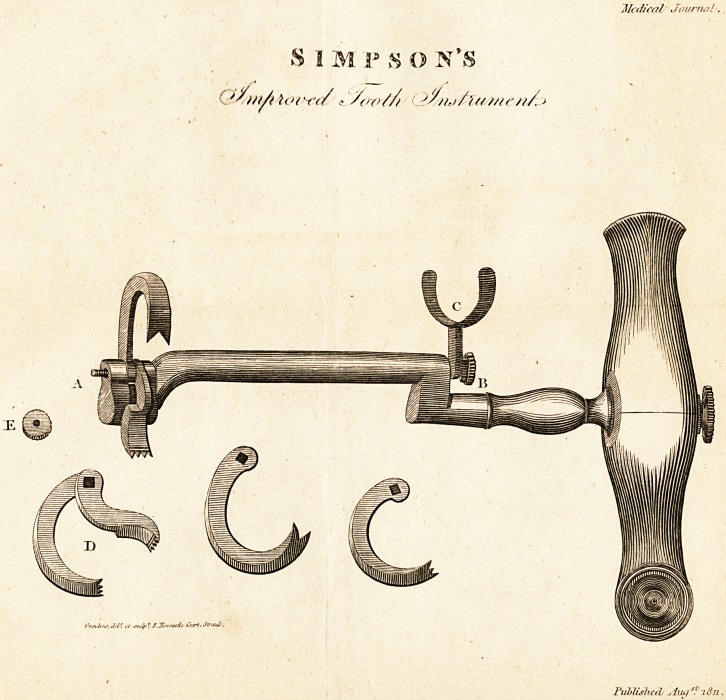# Medical and Philosophical Intelligence

**Published:** 1811-08

**Authors:** 


### MEDICAL AND PHILOSOPHICAL INTELLIGENCE.

### Description of Simpson's Tooth Instrument.

(With an Engraving.)

A B, two projecting extremities of a spindle which goes through the shank of the instrument, the part on which the two claws operate being perfectly square, the hole of the lower claw being round and large enough to admit of the free action of the spindle. C, the finger or thumb piece being attached to the extremity of the spindle B, gives the operator complete command of the upper claw, and enables him to place it with certainty on the most difficult stump without the introduction of his fingers into the mouth of the patient; and does away every danger of bruising or lacerating the gum, as there is no pressure on any part but the tooth he wishes to extract. Should the operator in any case prefer the bolster, he can withdraw the lower claw by drawing back the finger piece C, it then becomes the usual instrument, with the advantage of the claw being under the command of the fore-finger or the thumb of the hand in which the operator holds the instrument. D, the two claws as they appear when taken from the instrument; E, a small screw for going on the extremity of the spindle A.

The instrument above described is invented by Mr. Simpson, an ingenious manufacturer in the Borough: we have seen it employed, and have ourselves had some experience of its facility in application, and precision in action. We believe it to possess decided advantages over any other instrument employed for extracting teeth; and do not hesitate to recommend it to the notice of the profession. Editors.

---

Royal Society, May 20.—The reading of Mr. Travers' paper was concluded. It consisted of a summary of his experiments on wounds made into the cavity of the body, as it has been called. By these it appeared that the part denominated a cavity is always so extremely full, that no extravasation can take place in consequence of an horizontal or longitudinal puncture of the intestines, as in one case the lips of the wound are closed by pressure and cohesion, and in the other by inflammation.

The 13th of June, an account was read of a fœtus having been taken from the body of a woman, where it had remained 52 years. The narrative was written by Dr. Chester, who examined the body after death. The woman was a native of Gloucester, had been taken in labour as usual, but owing to the unskilfulness* of the midwife was not delivered. A surgeon was sent for; but when he arrived, the action of the uterus had subsided; in a few days the woman got well, and lived to the age of eighty, without having been delivered of the fœtus, when she died of

---

* It is probable this singular termination of labour was not influenced by the unskilfulness of the midwife, as the reporter states, but depended on a circumstance she might not foresee, nor if she had, could she have obviated.

paralysis.

paralysis. Dr. Chester having learned the history of the case, opened the body, and found an ossified globe which contained the perfect child, the arms and legs of which were somewhat compressed by this osseous mass, and in some parts absorption had taken place. The foetus was livid, but not putrid: the bony shell in which it was enveloped was thick and hard. This report (Phil. Mag.) is defective, inasmuch as it does not state the cavity in which the osseous mass was found. It was, probably, external to the uterus in the cavity of the abdomen, and was, perhaps, an extra-uterine foetation, or one of those cases which arise from retroverted uterus of the latter period of pregnancy, which have been so clearly explained by Dr. Merriman.

A paper on the *alcohol* of wine was read by Mr. Brande. The object was to refute or confirm the opinion of Fabrioni, that *alcohol* is a product of distillation, and not an essential part of the vegetable liquor. He gave a table of the quantity of *alcohol* contained in various wines and malt liquors; the highest was, that of Marcella wine, which contained 26 per cent of alcohol; red Champagne, 20; Port, from 20 to 24; Madeira, 19; Claret, 15; Cider and Perry, 12; Ale, 9; Brown Stout, 8; Porter, 6.

---

Royal Society of Edinburgh.—March 4, Mr. Allan read a paper on the rocks of the environs of Edinburgh, being the first of a series which he proposes to read on the subject. The present embraced the rocks of St. Leonard's Hill and Salisbury Craig. April 1, Dr. Brewster read a description of an instrument for measuring capillary attraction.

---

Wernerian Society.—April 27, Prof. Jameson read a paper concerning the geognostic relations of the Iceland crystal. The Secretary communicated an account of the *Colymbas Immer*, or Ember Goose, by Dr. Edmonston, of Shetland. Dr. Gordon read an interesting paper, consisting of observations and experiments on the qualities and sensations of sound; on the different modes in which sonorous vibrations are communicated to the auditory nerve; on the idea of the distance, and of the angular position of sounding bodies with respect to the ear, which are assosiated by experience with the different qualities of sound; and on some of the more remarkable differences in the *Sense of Hearing*, both original and accidental, which are occasionally observed among individuals, and in particular, on the musical ear.

---

Royal Medical Society of Edinburgh.—This Society will give a set of books, or a medal of five guineas value, to the author of the best essay in answer to the following question.

"*Does any decomposition of Acids and Alkalies take place in their uniting to form neutral salts, according to an opinion advanced by Mr. Davy in respect to the Muriates?*"

The Dissertations are to be written in English, Latin, or French, and to be delivered to the Secretary on or before the 1st of December, 1812. To each Dissertation must be affixed a *motto* to be written also on the outside of the sealed packet, containing the name and address of the author. No Dissertation will be received with the author's name affixed affixed; and all Dissertations, except the successful one, will be returned if desired, with the sealed packet unopened.

The adjudication of the *prize* will take place in the last week of February 1812.—Honorary, extraordinary, and ordinary Members of the Society are alone invited as candidates.

---

HARVEIAN SOCIETY OF EDINBURGH.—This Society resuming its accustomed plan of giving a copy of the 4to. edition of Harvey's works, published by the College of Physicians of London, for the best Dissertation on a subject proposed by the Society, has published the following *Questions* for competition.

For this year, 1811. *An Experimental Analysis of Diabetic Urine.*

For the year 1812. *An Experimental Essay on the best method of preparing a Soporific Medicine from the Lactuca Sativa.*

For the year 1813. *An Experimental Essay on the Effects of the Succus Spissatus Lactucæ sativa on the Human Body.*

Dissertations on the subject for 1811 must be transmitted to Dr. Duncan, senior, Edinburgh, principal Secretary to the Society, on or before the first of January, 1812. Each Dissertation must be accompanied with a sealed letter containing the name and address of the author, and marked on the back with a particular motto. The same motto must be prefixed to the Dissertation to which the letter belongs.

---

*Royal Society of Harlem*, continued from p. 82.—The following question was proposed for answer before the 1st of January, 1813. " Un catalogue exact des mammiferes, des oiseaux, et des amphibies qui, n'étant pas des especes transportées d'ailleurs, se trouvent naturellement dans ces pays-ci, contenant leurs différens noms en différentes parties de ce royaume, et leurs caractères génériques et spécifiques, décrits en peu de mots suivant le système de Linné, avec indication d'une ou plusieurs des meilleurs représentations de chaque animal ?"

The Society then proceeded to determine upon the answers to the questions relating to natural and moral philosophy, which had been proposed for the year 1809; and after rejecting some, and rewarding others, with the appropriate medal, it offered additional prize-questions on these subjects for 1812.

The following day, May 20th, the Society renewed its sitting, and determined upon the answers to questions in literary and antiquarian sciences.

The following questions continue to be proposed for an unlimited time.

I. " Qu'est-ce que l'expérience a appris concernant l'utilité de quelques animaux qui sont en apparence nuisibles, surtout dans les Pays-Bas, et quelles précautions doit-on observer à l'égard de leur extirpation ?"

II. " Quelles sont les plantes indigènes le moins connues jusqu'ici par leur vertu, que l'on pourrait employer avec utilité dans nos pharmacopées, et qui pourraient remplacer les remédes exotiques ?"

III. " De quelles plantes indigènes, qui ne sont pas en usage jusqu'ici, pourrait-on se servir pour une bonne nourriture et à bas prix ; et quelles plantes nourrissantes exotiques, ou qui se trouvent dans d'autres pays, pourrait-on cultiver ici dans le même but ?"

IV. " Quelles plantes indigènes, qui sont inusitées jusqu'ici, peuvent, d'après des expériences bien confirmées, donner de bonnes couleurs, dont la préparation et l'usage pourrait être introduit avec profit; et quelles plantes exotiques pourrait-on cultiver avec profit dans des terres moins fertiles ou peu cultivées de cette république, pour en extraire des couleurs?"

V. "Que sait-on actuellement du cours ou de mouvement de la sève dans les arbres et dans les plantes? De quelle manière pourrait-on acquérir une connoissance plus complette de ce qu'il y a encore d'obscur et de douteux à cet égard?"

The Society repeats, that it determined at the anniversary meeting in 1798 to consider at each subsequent anniversary, whether any of the papers transmitted to it (not being answers to prize-questions) on subjects of natural history or natural philosophy, merit a particular gratification; in which case it will be rewarded with a silver medal, and ten ducats. Authors are requested to make their communications as short as the nature of the subject will permit.

Answers to the prize-questions may be written (in *Italic* characters) in Dutch, French, Latin, or in German; they must appear to be written in the author's own hand-writing; his name and address must be given in a sealed note, and forwarded to M. Van Marum, perpetual Secretary to the Society.

## Figures and Tables

**Figure f1:**